# The first comorbidity networks in companion dogs in the Dog Aging Project

**DOI:** 10.1101/2024.12.18.629088

**Published:** 2024-12-20

**Authors:** Antoinette Fang, Lakshin Kumar, Kate E Creevy, the Dog Aging Project Consortium, Daniel E.L. Promislow, Jing Ma

**Affiliations:** 1. Department of Mathematics, University of Chicago; 2. Department of Medicine, University of California, San Francisco; 3. College of Veterinary Medicine & Biomedical Sciences, Texas A&M University; 4. Jean Mayer USDA Human Nutrition Research Center on Aging, Tufts University; 5. Division of Public Health Sciences, Fred Hutchinson Cancer Center

## Abstract

Comorbidity and its association with age are of great interest in geroscience. However, there are few model organisms that are well-suited to study comorbidities that will have high relevance to humans. In this light, we turn our attention to the companion dog. The companion dog shares many morbidities with humans. Thus, a better understanding of canine comorbidity relationships could benefit both humans and dogs. We present an analysis of canine comorbidity networks from the Dog Aging Project, a large epidemiological cohort study of companion dogs in the United States. We included owner-reported health conditions that occurred in at least 60 dogs (n=166) and included only dogs that had at least one of those health conditions (n=26,523). We constructed an undirected comorbidity network using a Poisson binomial test, adjusting for age, sex, sterilization status, breed background (i.e., purebred vs. mixed-breed), and weight. The comorbidity network reveals well-documented comorbidities, such as diabetes with blindness and hypertension with chronic kidney disease. In addition, this network also supports less well-studied comorbidity relationships, such as proteinuria with anemia. A directed comorbidity network accounting for time of reported condition onset suggests that diabetes occurs before cataracts, which is consistent with the canine literature. Analysis of age-stratified networks reveals that global centrality measures increase with age and are the highest in the Senior group compared to the Young Adult and Mature Adult groups. Our results suggest that comorbidity network analysis is a promising method to enhance clinical knowledge and canine healthcare management.

## Introduction

Comorbidity refers to the simultaneous presence of two health conditions in an individual organism [1,2]. The understanding of comorbidities can offer insights into health condition associations and progression, which can in turn improve predictive and diagnostic tools in the clinic [3–6]. The strong positive association between measures of comorbidity and age has led those in the geroscience community to explore the mechanisms by which comorbidity might be a cause or consequence of aging [7–11]. Comorbidities are often studied in the form of a network [12–18], where each node in the network represents a health condition and each edge represents a statistically significant association between a pair of health conditions. Many health conditions are related through a variety of biological mechanisms, and comorbidity networks can help us better understand these underlying connections. Thus far, there are several large studies that use a variety of methods investigating comorbidities and comorbidity networks in humans [14,15,18–21]. The same is not true for dogs, for which there is a limited understanding of comorbidities. Notably, dogs are a good model organism because they share the same environments and many of the same health conditions as humans, they have an almost equally sophisticated healthcare system, and show similar manifestations of aging as humans [22]. Existing dog comorbidity studies are limited in sample size and diversity [23–26], or focus on comorbidities in relation to an index health condition [26–30]. While some previous studies controlled for demographic covariates when examining dog comorbidities [26], many studies either do not report adjusting for such variables or do not explicitly describe their methods for handling dog demographics and other important covariates [23–25,27–30].

In this study, we address these limitations by constructing a comorbidity network for more than 150 canine morbidities using data from a large cohort (n=26,259) assembled by the Dog Aging Project (DAP) [22]. The DAP is a long-term longitudinal study of the genetic, environmental and lifestyle determinants of healthy aging in companion dogs. As of November 2024, more than 50,000 participants across the United States have signed up their dogs. Participating dog owners fill out an annual survey that includes extensive information on both owner demographics and dog signalment, the home and external environment, and the dog’s physical activity, behavior, diet, medications, and of direct relevance to the present study, health status. Having completed this first survey, the dog becomes a member of the DAP “Pack” cohort, and all participants are invited to continue to provide additional data about their dogs over time. Through analysis of owner-reported health data, we identify both well-documented and novel health condition associations, demonstrate age-dependent patterns in health condition co-occurrence, and investigate temporal relationships between health conditions. These findings contribute to veterinary informatics and health policy, expanding opportunities for evidence-based medicine in veterinary practice.

## Results

### Study cohort

We included health conditions that occurred in at least 60 dogs, and we only included dogs that had at least one of these conditions. The final cohort encompasses 166 unique health conditions and 26,259 dogs, each with at least one of the 166 conditions (Table 1). The cohort has an approximately equal proportion of male and female dogs, but only 7% of dogs are reproductively intact (unspayed/unneutered). Also, there are roughly equal numbers of purebred and mixed-breed dogs. Dogs in this cohort have a median age of 7.8 years and median weight of 50.8 lbs. The health conditions are grouped into 20 body system categories. The skin category contains 21 health conditions (the highest number), while some other categories (congenital, immune-mediated, and hematopoietic) contain only one health condition (Table S1a). Additionally, most dogs have three or fewer health conditions, with just 37% of dogs having four or more health conditions (Fig. S1).

### Association between demographic factors and health conditions

The coefficients in the logistic regression models provide insights into factors associated with different health conditions that are intuitive and consistent with previous findings. For example, over 80% of the models in which the age coefficient was statistically significant (P < 0.05) had a positive age coefficient (Table S2). This supports the notion that older age is an important risk factor for many health conditions [31]. Conditions that had a significant negative coefficient for age were conditions we would expect to occur in younger dogs, such as retained baby teeth, cryptorchidism, or coccidia [32]. Weight (as a measure of body size) showed significant associations with approximately 68% of health conditions, with 57% of these significant associations being positive. This supports previous research showing that some conditions are more prevalent in larger dogs, while others occur more frequently in smaller dogs [33,34]. The sex variable coefficients were not statistically significant for over 90% of the conditions [34]. There were several health conditions for which correlation coefficients differed by sex. However, these health conditions also tend to be those that affect the reproductive organs of male or female dogs (e.e.g., cryptorchidism or pyometra) [32]. The breed background coefficient was only significant for around half of the models, and of those, less than 40% were positive (i.e., greater in purebred dogs). Purebred dogs showed significantly higher rates of ear infection, cataracts, and intervertebral disk disease. In comparison, mixed-breed dogs had significantly higher rates of cruciate ligament rupture, being hit by a car, pruritis, and hookworms. No significant differences were found between purebred and mixed breed dogs for conditions such as hip dysplasia, patellar luxation, hypoadrenocorticism, hyperadrenocorticism, and mast cell tumors, aligning with findings from previous work [35,36].

### Undirected comorbidity network

**Figure 1.**
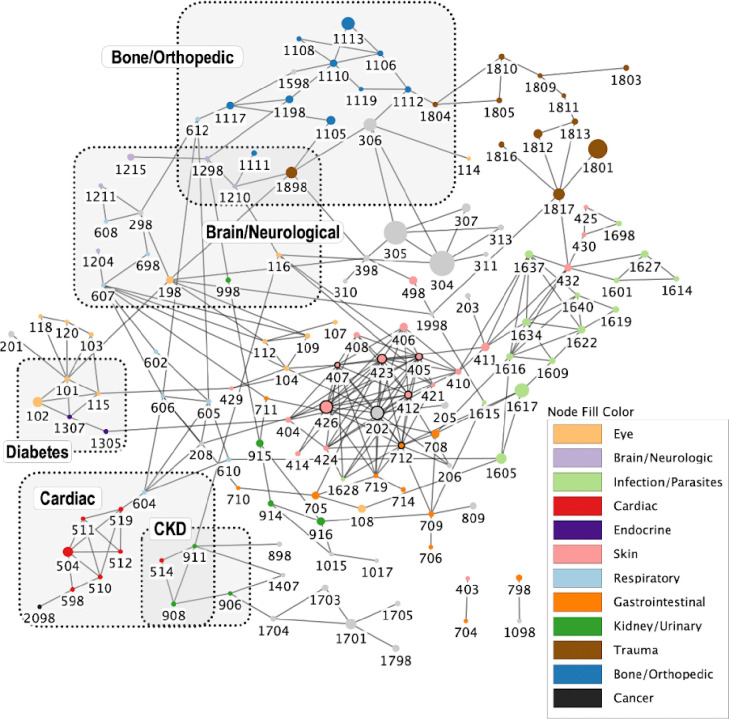
Undirected comorbidity network. Nodes are health conditions, and edges are statistically significant comorbidity connections (at a Bonferroni adjusted P-value < 0.001). Node sizes represent lifetime prevalence, with more common health conditions having larger nodes. Nodes with degrees greater than 10 are outlined in bold. Additionally, several health conditions or health condition groups of interest in geroscience, as outlined by the National Institute of Aging, as well as their first-order neighbors are highlighted with shaded boxes [44]. These health condition groups as well as the five health condition categories with the highest number of affected dogs have colored nodes. All other categories have gray nodes. Health condition categories were created using the categorization system in the DAP survey and the majority of the categories highlighted in the network can best be described as organ systems (i.e., respiratory, cardiac) as opposed to pathophysiological processes (i.e., trauma, infection) [45]. Refer to Table S1b for the corresponding health condition name associated with each numeric code.

The undirected comorbidity network is presented in Fig. 1. A complete table of all health condition pairs and their corresponding P-values, regardless of statistical significance, is provided in Table S3a. Of note, health conditions of the same categories tend to be connected with each other. This network models a variety of well-documented comorbidities: diabetes (node 1307) and blindness (node 101) (P < 0.001); atopic dermatitis (node 405) and allergies (nodes 412) (P < 0.001); and Cushing’s disease (node 1305) and alopecia (node 404) (P < 0.001) [32]. All of these pairs include eye or skin health conditions, and these health conditions are commonly used by veterinarians as indicators of systemic diseases like diabetes [32,37]. In addition to these skin- and eye-related health condition pairs, we also see some other well-known health condition pairs like hypertension (node 514) and chronic kidney disease (CKD, node 908) (P < 0.001) [38,39]. The network demonstrates the central role of ear infections (node 202) with a high degree of connectivity (11 connections), linking it to multiple dermatological conditions like atopic dermatitis or chronic and recurrent skin infections (nodes 405 and 407); various forms of allergies like food/medicine allergies affecting the skin or seasonal allergies (nodes 412 or 426); and other complications like hearing loss and keratoconjunctivitis sicca (nodes 205 and 104). This pattern of connectivity quantitatively supports clinical observations that ear infections often occur as part of a broader pattern of allergic and inflammatory conditions, particularly those affecting the skin [40]. The multiple connections to allergic conditions align with previous findings that hypersensitivity diseases are among the most common primary factors leading to otitis in dogs [40]. The network also reveals a notable cluster of parasite and parasite-related health conditions, which supports the observation that parasites often co-occur [41–43].

In addition to well-known comorbidities, this network also supports less well-studied health condition pairs. For example, this network suggests an association between proteinuria (node 911) and anemia (node 1407) (P <0.001). Proteinuria describes the presence of protein in the urine and may be associated with acute or chronic kidney disease, as well as with certain systemic health conditions [46]. Anemia, a deficiency of red blood cells, is also often present in advanced CKD due to the combination of impaired red blood cell survival deficiency of production of erythropoietin by the kidney, and impaired bone marrow responsiveness to erythropoietin [47–49]. The network illustrates these relationships by connecting anemia to CKD (node 908) through proteinuria. While our undirected network cannot imply causal relationships or mediation effects, it does highlight potential associations that warrant further investigation. A recent study conducted with a small cohort of 37 dogs suggests that proteinuria may contribute to anemia in dogs with CKD, a relationship that has also been suggested in other model organisms [49–51]. The connections revealed by our network demonstrate its potential in identifying less obvious health condition associations and comorbidities.

Next, we examined the node degree distribution to see if the network can be classified as random or scale-free. Node degree, which is defined as the number of edges connected to each node [52], is one measure of node centrality. The degree distribution is one of the most fundamental properties of networks and provides important information about a network’s structure [53]. The node degree distribution of our network is better fitted with an exponential distribution than a scale-free power-law distribution (P < 0.001) (Fig. S2), providing evidence for a random network.

### Comparison to stratified networks

**Figure 2.**
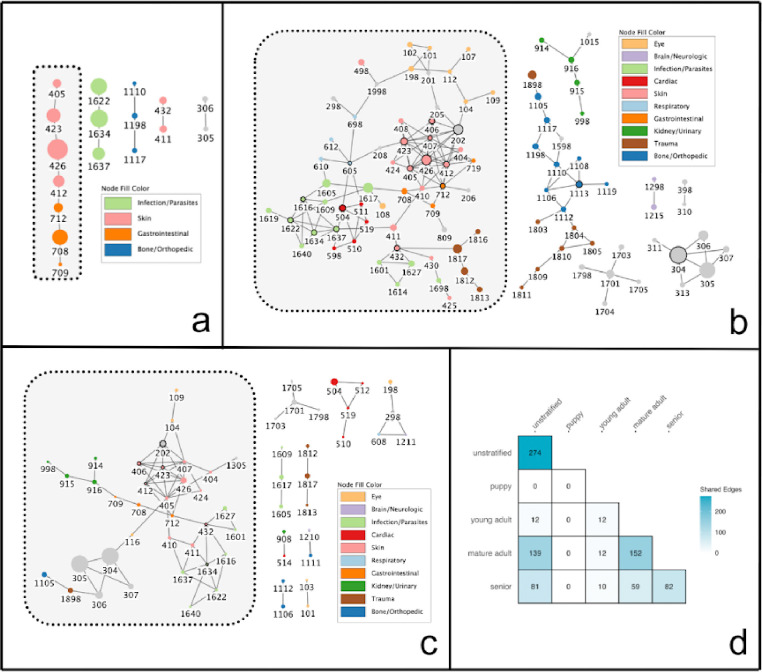
Age-stratified comorbidity networks. (a) Young Adult, (b) Mature Adult, and (c) Senior dog networks. In each network, nodes represent health conditions with node size proportional to condition prevalence, and edges represent statistically significant comorbidity associations (Bonferroni-adjusted P < 0.01). Nodes with the highest degree in each network are indicated with bold outlines, and the largest connected subnetwork is highlighted with a dotted shaded box. No network is shown for the Puppy stratum as no significant comorbidities were detected in this age group. (d) Heatmap displaying edge overlap between disease networks across age groups. Numbers indicate shared edges between a pair of networks, with diagonal values showing total edges per network.

We stratified the dataset into four groups by life stage (Puppy: n=158; Young Adult: n=3487; Mature Adult: n=15784; Senior: n=6119) to investigate the extent to which comorbidity networks varied across life stages. As expected, the four networks constructed using the stratified approach differed greatly from each other and also from the unstratified network (Fig. 2). Notably, no significant comorbidity patterns were detected for the puppy age group, which comprised only 2% of the cohort. This likely reflects the relatively low frequency of multiple health conditions in young dogs rather than simply a statistical power limitation.

Ten comorbidity pairs were identified across all four networks: roundworms and tapeworms (nodes 1634 and 1637), hookworms and roundworms (nodes 1622 and 1634), chronic/recurrent diarrhea and gastrointestinal food/medicine allergies (nodes 708 and 712), chronic/recurrent diarrhea and chronic/recurrent vomiting (nodes 708 and 709), atopic dermatitis and pruritus (nodes 405 and 423), pruritis and seasonal allergies (nodes 423 and 426), extracted teeth and fractured teeth (nodes 305 and 306), skin food/medicine allergies and gastrointestinal food/medicine allergies (nodes 412 and 712), skin food/medicine allergies and seasonal allergies (nodes 412 and 426), and fleas and ticks (nodes 411 and 432). Conversely, there were 14 unique comorbidity pairs identified by the stratified networks but not by the unstratified network. Ten of these pairs came from the mature adult group and two from the senior group (Fig. S4). On the other hand, the unstratified network identified 113 comorbidities that were not detected by *any* of the stratified networks. However, this could be due to lower statistical power in the stratified analysis.

Network topology analysis of the age-specific networks revealed distinct patterns in disease interconnectedness (Table 2). Edge density displayed a different trajectory than other measures, where it was the highest in the young adult network. By contrast, all other measures– clustering coefficient, betweenness centrality, and degree centrality– increased with age. This suggests a progression from a less structured and more diffuse network in the young adult life stage to an increasingly modular network with a few hub nodes as dogs age. Together, these results support the idea that age is a critical factor influencing comorbidity development.

### Adding temporal P-values to understand health condition progression

**Figure 3.**
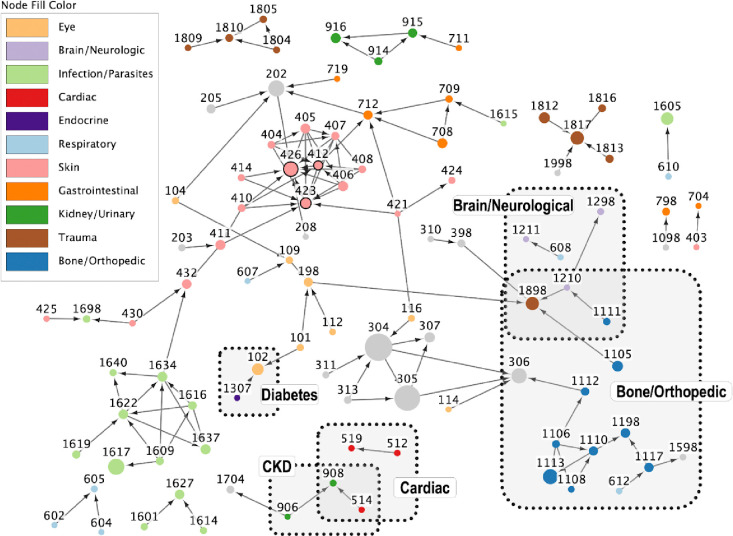
Time-directed network using a window size of 12 months. Nodes are health conditions, and edges are statistically significant comorbidity connections (at a Bonferroni-adjusted P-value < 0.01). Arrowheads point from the health condition that occurs earlier in time to the health condition that occurs later. Node sizes represent prevalence, with more common health conditions having larger nodes. Nodes with total node degree greater than 10 are outlined in bold. Additionally, several health conditions or health condition groups of interest in geroscience, as well as their first-order neighbors are highlighted with shaded boxes [44]. These health condition categories as well as the top five best-represented health condition categories also have colored nodes. All other categories have gray nodes. Refer to Table S1b for the corresponding health condition name associated with each numeric code.

Finally, we examined temporal relationships between conditions by computing the probability of one condition preceding another within a time window of 12 months and testing its significance using the Poisson binomial approach to comorbidity (see Methods for details). A time-directed comorbidity network is presented in Fig. 3. This makes the network more informative, as the directed network gives suggestions about health condition progression. For instance, the directed network suggests that diabetes (node 1307) occurs before cataracts (node 102) (P < 0.001). This is consistent with the literature: it is estimated that around 75% of canines diagnosed with diabetes will have blinding cataracts within the following two years [54]. Other examples of directional pairings supported by existing literature can be found in Fig. S5.

As in the undirected network (Fig. 1), the directed network also exhibits a cluster of parasite and parasite-related health conditions. However, the mechanisms of multiparasitism, especially in companion dogs, are poorly understood because previous work focuses on a one host-one parasite paradigm [42]. The addition of temporal information in this network could support future research endeavors in this area.

## Materials and Methods

### Dog Aging Project owner survey data

The DAP is a nationwide, long-term, longitudinal study on the biological, environmental, and lifestyle determinants of healthy aging in companion dogs [22]. All data are made publicly available (other than identifiable owner information) through the Terra platform at the Broad Institute at MIT and Harvard. Data on signalment, husbandry and health status of the dogs in the study are collected from all participants through a comprehensive owner-reported survey; subsets of participants provide other types of information as well. The Health section of the baseline Health and Life Experiences Survey (HLES) captures lifetime prevalence of health conditions, defined as the proportion of individuals in a population who have experienced a particular condition or event at any point in their lives up to the time of the survey or study. Owners follow survey logic to identify their dogs’ health conditions or clinical signs, organized by process (e.g., trauma) or body system (e.g., cardiac). Owners are presented with more than 300 different health conditions or clinical signs, with an option for “other, please describe” in each body system, and also provide the time of onset for each reported condition. This study utilized the 2021 DAP Curated Data Release, which contains all survey responses obtained by 12/31/2021. As such, while the data we analyze are technically cross-sectional in nature because they were reported at a single point in time, the survey items are constructed to capture lifetime prevalence as defined above.

Of note, the following information from this dataset was used: (1) age, sex, sterilization status, breed background, and weight of each dog, and (2) list of health conditions (out of 365 possible, distinct health conditions, Table S1b) and the date of reported condition onset (if any) for each dog. Note that the list of health conditions here reflects the health condition options presented to dog owners on the survey.

### Logistic regression modeling

To estimate the individualized probabilities for each health condition, we fit logistic regression models (LRM). Previous veterinary work suggests that factors such as age, sex, spay/neuter status, weight, and breed background are associated with health condition development in dogs [31,33–35,55,56]. Thus, each LRM included the following demographic characteristics as covariates: age, weight, sex, and whether the dog is purebred or mixed-breed. Sex is defined as a four-category variable (intact female, spayed female, intact male, neutered male). Thus, there are in total six predictors in each LRM. We used breed background as opposed to breeds as a covariate because there are a large number of breeds and many health conditions are very rare. Fitting LRMs on rare events data with a large number of covariates can lead to large bias in estimation and statistical inference [57]. Despite being an imprecise summary, breed background has previously been reported to be associated with several health conditions [35]. The response variable is binary indicating whether the dog has a given health condition. The LRMs estimate each dog’s probability for having a certain health condition $P_{y\in d}$:

(1) $$P_{y\in d}=\frac{1}{1+exp\left(-\left(\beta_{0}+\beta_{1}x_{1}+\ldots +\beta_{6}x_{6}\right)\right)}.$$

### Comorbidity P-values

The null hypothesis is that a pair of health conditions y,z are independently distributed: $H_{0}=y⊥z$. A significant P-value indicates evidence that the health condition pair co-occur more often than by chance. Previous canine comorbidity literature largely relies on methods that assume that the probability of seeing health conditions y,z in a dog $P_{\{y,z\}\in d}$ is equal to the product of the population incidence rate for each health condition under the null hypothesis:

(2) $$P_{y\in d}=\frac{C_{y}}{N},P_{z\in d}=\frac{C_{z}}{N}$$

(3) $$P_{\{y,z\}\subset d}=\frac{C_{y}}{N}\times \frac{C_{z}}{N}$$

where $C_{y}$, $C_{z}$ represents the prevalence of health conditions and N the total number of dogs. A comorbidity P-value can then be calculated under the binomial distribution. However, this method does not account for important covariates that could impact individual disease risks. A standard approach to deal with covariates is to stratify the population into homogeneous subgroups [15,18,19]. The downside of this approach is that it greatly decreases statistical power [18]. To overcome this issue, we used the Poisson binomial approach to comorbidity (PBC) [18] method to calculate P-values for each health condition pair. We opt for the PBC method as opposed to other notions of health condition associations [16], because PBC does not require sample stratification and has been shown to have higher power in comorbidity discovery [18]. The probability that two health conditions occur by chance in a dog is simply the product of the two individualized health condition probabilities given by the LRMs described previously:

(4) $$P_{\{y,z\}\subset d}=P_{y\in d}\times P_{z\in d}.$$

In short, the Poisson binomial distribution is a generalization of the binomial distribution in which every dog has its own probability of having a health condition. Since the cumulative distribution function for the Poisson binomial is computationally unwieldy, we used the normal approximation, which is accurate but also faster [18,58]. To determine a P-value approximately, we estimated the mean and variance under the normal distribution as follows. The mean in this case represents the expected number of dogs with both health conditions:

(5) $$\mu_{y,z}=\sum_{d}P_{\{y,z\}\subset d}.$$

The variance is

(6) $$\sigma_{y,z}^{2}=\sum_{d}\sigma_{P_{\{y,z\}\in d}}^{2}=\sum_{d}P_{\{y,z\}\subset d}\left(1-P_{\{y,z\}\subset d}\right).$$

Initial analyses showed that accounting for the uncertainties in the predicted individual probabilities through the delta method did not meaningfully alter the comorbidity relationships identified. Therefore, we proceed with the variance presented in Equation (6).

Finally, as described previously, we can find the comorbidity P-values using the normal approximation of the Poisson binomial distribution. Health condition pairs with Bonferroni-adjusted P<0.001 were included in the final network. Comorbid pairs were also verified by finding the Pearson correlation coefficient for the individualized health condition probabilities for each pair. Only pairs with positive coefficients were included.

### Reported onset dates and directed networks

We extended our analysis to examine temporal relationships between conditions by analyzing the owner-reported dates when each condition first occurred in their dog. For each pair of health conditions y, z, we analyzed the temporal relationship using only dogs reported to have both conditions, comparing their individual dates of reported condition onset to determine which condition manifested first. Unlike population-level approaches that compare average age of condition onset across all cases, our method examines the sequence of reported conditions within individual dogs’ health histories. This tells us whether a health condition is more likely to occur before another health condition. The probability that the reported onset of health condition y occurs before the reported onset of health condition z in a dog that has both conditions y, z is

(9) $$P_{y\to z}^{\{y,z\}\subset d}=\frac{P_{y\in d}}{P_{y\in d}+P_{z\in d}}.$$

We also incorporated a time window size W and the length of the dog’s medical history as done in Lemmon *et al*. [18]. The difference between each dog’s most recent date of reported condition onset and earliest date of reported condition onset was used as a proxy for its medical history span (Fig. S3). Knowing the span of each dog’s medical history allows us to study directed comorbidities across prespecified time windows. Here we chose a window of 12 months. Now, the probability that y, z occur within W given that the canine already has the two health conditions is:

(10) $$\begin{array}{c} P_{\{y,z\}\subset d\;\mathrm{in}\;W}=\frac{W}{\mathrm{Span}}\left(2-\frac{W}{\mathrm{Span}}\right)\;\mathrm{if}\;\mathrm{Span}>W,\\ P_{\{y,z\}\subset d\;\mathrm{in}\;W}=1\;\mathrm{if}\;\mathrm{otherwise}. \end{array}$$

We combined these two probabilities to get the probability that y occurs within W before z as:

(11) $$P_{y\to z\;\mathrm{in}\;W}^{\{y,z\}\subset d}=P_{y\to z}^{\{y,z\}\subset d}\times P_{\{y,z\}\subset d\;\mathrm{in}\;W}.$$

Finally, as described previously, we can find the direction P-values using the normal approximation of the Poisson binomial distribution. Note that for each health condition pair y,z, we calculated both $P_{y\to z\;\mathrm{in}\;W}^{\{y,z\}\subset d}$ and $P_{z\to y\;\mathrm{in}\;W}^{\{y,z\}\subset d}$. Health condition pairs with Bonferroni adjusted P < 0.01 were included in the final network.

### Comorbidity network with stratification

We stratified the DAP dogs into four life-stage cohorts (from youngest to oldest): puppy, young adult, mature adult, and senior. These life stages were constructed using DAP veterinary guidelines that incorporate weight, breed, and sex in addition to age [59]. Within each stratum, we selected health conditions that at least 60 dogs had and included only dogs with at least one of those selected health conditions, similar to the criteria for the unstratified network. We again used the PBC method described previously to identify potential comorbidity relationships within each age stratum. Health condition pairs with Bonferroni adjusted P < 0.01 were included in the final networks, with one network for each stratum (Fig. S2).

## Discussion

This paper leverages data from the DAP, which encompasses a large epidemiological cohort, to study the canine comorbidity network among 166 different health conditions. Key demographic characteristics including age, weight, sex, sterilization status, and breed background were adjusted for using LRMs before constructing the comorbidity network. Our LRMs support the contribution of key demographic characteristics to the risk of health conditions [31,33–35,55,56].

The undirected canine comorbidity network reveals both well-known comorbidities and suggests potential connections between health conditions that have been less studied [32,37–39,46,49]. This illustrates the potential for using health condition association networks to aid with novel comorbidity discovery. The network’s degree distribution is better modeled with an exponential distribution when compared with a scale-free power-law distribution. Had we observed support for the power-law distribution, this could have been interpreted as suggesting that the risk of having an additional morbidity is directly proportional to the number of morbidities a dog already has. By contrast, the exponential distribution is consistent with a model where the probability of developing a morbidity is independent of the number of morbidities a dog already has. However, we note that since this is a small network, observing a power-law degree distribution was unlikely to begin with [60,61].

When comparing the unstratified network to the four networks created after stratification by life stage, the unstratified network identifies many associations not identified by stratified networks. This makes sense due to the reduction in statistical power after stratification. On the other hand, stratification revealed important age-specific patterns. For instance, within the stratified networks, only the senior network identified comorbidities in the cardiac and neurological categories. Both categories are of particular interest in aging research. This suggests that while the unstratified network is valuable for broad discovery, combining it with stratification can provide further insights when focusing on specific conditions or specific groups of health conditions. Such a combined approach allows for a comprehensive understanding of both overall comorbidity patterns and age-specific associations.

Finally, we also constructed a time-directed network using a window size of one year. Shorter window spans are more adept at capturing comorbidities that occur sequentially [1], but could result in power loss in comorbidity discovery since shorter window sizes encompass fewer dogs. The ideal window size should be guided by clinical knowledge and research objectives [18]. Incorporating the time of reported condition onset provides us with some insights into health condition progression, which could be beneficial for canine health management. For instance, this time-directed network exhibits a cluster of parasite-parasite interactions. While interest in co- or multiparasitism has increased in recent years, more work is needed to understand the mechanisms of these interactions [41–43,62,63]. The temporal information encoded in the directed network may suggest potential synergistic relationships between parasites [42]. Understanding and controlling companion dog parasitism is becoming increasingly important as pet ownership, particularly of dogs, continues to rise globally [64]. This growing human-animal interface elevates the significance of our findings beyond veterinary medicine, potentially informing public health strategies related to zoonotic health conditions [64–66].

There are a few limitations in this study. First, the use of owner-reported lifetime prevalence collected at a single point in time introduces risk of recall bias. Owners may not accurately recall all of the dog’s health conditions, or the age at occurrence of each condition. This limitation will be overcome with time as the DAP continues longitudinal data collection; repeating the survey-based inventory of health conditions each year for each dog shortens the interval required for owner recall to one year. Second, rare health conditions are susceptible to overfitting by LRMs, which may result in an inaccurate representation of comorbidity between rare and prevalent health conditions in the network. One example of this is pododermatitis (node 421, n=65), a skin condition, and allergies (node 412, n=1015). General clinical knowledge suggests that allergies are a common cause of pododermatitis [67]. However, this pairing is not observed in the network. Third, owners may misreport health status information. The list of health conditions in the DAP surveys was designed to make the survey easier for owners to complete, so some of the health conditions may overlap or are medically imprecise. Also, health condition categories better align with organ systems, so a single pathological process that is implicated in multiple systems could end up being described in the survey as two or more health conditions. This is directly observed in the network as there are several instances where one pathological process is instead represented as a comorbid pair, like laryngeal paralysis duplicated in both respiratory and brain/neurological conditions (nodes 608 and 1211). Beyond general recall accuracy, the temporal sequence of conditions may be particularly challenging for owners to report accurately, as concurrent or gradually developing conditions can be difficult to order chronologically. The owner-reported dates of condition onset do not necessarily represent the pathophysiological course of a health condition, or may themselves be inaccurate. This issue likely explains some counterintuitive results like tick-borne health conditions preceding ticks in the time-directed network (Fig. 4). Fourth, veterinarians may only recommend, or owners may only elect to proceed with, certain diagnostic tests based on prior knowledge of a dog’s current diagnoses, which could introduce confirmation bias into the network. For instance, blood pressure testing is likely to be recommended in dogs with diagnosed kidney disease, due to the known association of hypertension with kidney disease. However, in dogs without kidney disease, blood pressure may not be measured at all, meaning that lack of owner knowledge/reporting of their dog’s hypertension is not equivalent to absence of hypertension in their dog. Furthermore, at least at the time of enrollment, dogs in the Dog Aging Project might be healthier than average if owners are disinclined to sign up a very unhealthy dog for a study on the determinants of healthy aging, similar to the well-known “healthy volunteer” effect in human studies [68–70]. Finally, the owners in our study cohort are predominantly white, highly educated, and wealthier than the US population, which may raise concern on the generalizability of the identified comorbidities (Fig. S6).

Acknowledging these limitations, this study demonstrates the power of the DAP data, which has great potential to uncover further insights. First, while this report focuses on positively correlated health condition pairs, our analysis also identified several negatively correlated pairs. A systematic investigation of these negative associations could reveal potential antagonistic relationships between conditions, though careful interpretation would be needed to distinguish true biological relationships from competing risk effects. Second, DAP has access to veterinary electronic medical records (VEMRs) that can be mined using natural language processing and analyzed using the network methods. It would be interesting to compare a network learned from VEMRs with the one inferred from owner-reported data. Furthermore, we have already collected genetic profiles for over 7000 dogs in DAP. Future work could use gene-relatedness learned from these genetic profiles to control for genetic differences among dogs, potentially surpassing the precision of our current binary classification of breed background. The inclusion of genetic profiles would better reflect the subtle but important differences between various dog breeds and help elucidate the molecular mechanism underlying canine comorbidities.

## Figures and Tables

**Table 1:** Demographic characteristics of the companion dogs included in the comorbidity network analysis.

Characteristic	N = 26,253^1^
*Sex*	
Female, spayed	12, 416 (46%)
Female, unspayed	679 (3%)
Male, neutered	12, 164 (46%)
Male, unneutered	1, 264 (5%)
*Breed*	
Mixed breed	13, 374 (50%)
Purebred	13, 149 (50%)
*Age (years)*	7.8 [4.1, 11.0]
*Weight (lbs)*	50.8 [25.0, 69.0]

1 n (%); Median [Q1, Q3]

**Table 2:** Network topology measures across life stages: edge density refers to the number of edges relative to the maximum number of possible edges in each graph; clustering coefficient measures the degree to which nodes in each graph cluster together; betweenness and degree centrality refer to the normalized graph level centralization measure based on the respective node-level centrality scores.

	Puppy	Young Adult	Mature Adult	Senior
**Edge Density**	N/A	0.088	0.032	0.043
**Clustering Coefficient**	N/A	0	0.473	0.484
**Betweenness Centrality**	N/A	0.0604	0.125	0.136
**Degree Centrality**	N/A	0.0417	0.0831	0.108

## Data Availability

This study uses data in the Health and Life Experiences Survey (HLES) from the 2021 DAP Curated Data Release. These data are housed on the Terra platform at the Broad Institute of MIT and Harvard. Access to these data can be applied for free at https://dogagingproject.org/data-access.
