# Supplementary figures and images for: Constructing the first comorbidity networks in companion dogs in the Dog Aging Project
Source: bioRxiv. 2025 Jul 11:2024.12.18.629088. Originally published 2024 Dec 20. Preprint. [Version 2] doi: 10.1101/2024.12.18.629088 (PMC11702704; doi:10.1101/2024.12.18.629088)

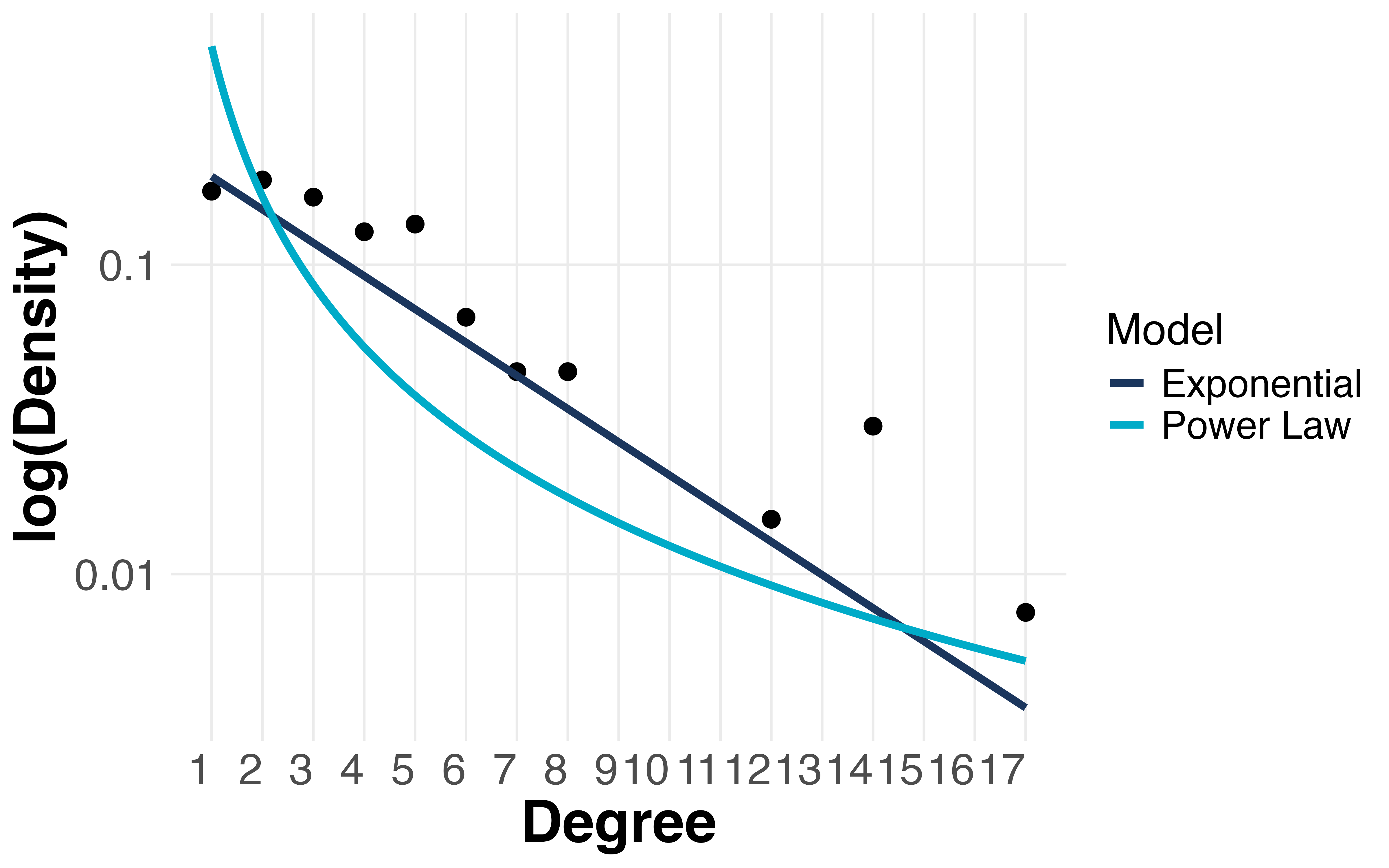

Supplement: Supplement 7 — S1 Fig. Log scale density distribution of node degrees in the undirected comorbidity network. The overlaid lines represent the density distribution predicted by an exponential and scale-free power-law model. [file media-7.tif]
